# A Recent Ten-Year Perspective: Bile Acid Metabolism and Signaling

**DOI:** 10.3390/molecules27061983

**Published:** 2022-03-18

**Authors:** Yulia Shulpekova, Elena Shirokova, Maria Zharkova, Pyotr Tkachenko, Igor Tikhonov, Alexander Stepanov, Alexandra Sinitsyna, Alexander Izotov, Tatyana Butkova, Nadezhda Shulpekova, Vladimir Nechaev, Igor Damulin, Alexey Okhlobystin, Vladimir Ivashkin

**Affiliations:** 1 Chair of Internal Diseases Propedeutics, Gastroenterology and Hepatology, Sechenov First Moscow State Medical University (Sechenov University), 119048 Moscow, Russia; shulpekova_yu_o@staff.sechenov.ru (Y.S.); shirokova_e_n@staff.sechenov.ru (E.S.); tkachenko_p_e@staff.sechenov.ru (P.T.); tikhonov_i_n@staff.sechenov.ru (I.T.); nechaev_v_m@staff.sechenov.ru (V.N.); okhlobystin_a_v@staff.sechenov.ru (A.O.); ivashkin_v_t@staff.sechenov.ru (V.I.); 2 Department of Hepatology University Clinical Hospital No.2, Sechenov First Moscow State Medical University (Sechenov University), 119048 Moscow, Russia; zharkova_m_s@staff.sechenov.ru; 3 Biobanking Group, Branch of Institute of Biomedical Chemistry “Scientific and Education Center”, 109028 Moscow, Russia; aleks.a.stepanov@gmail.com (A.S.); anvilya@gmail.com (A.S.); izotov.alexander.ibmc@gmail.com (A.I.); t.butkova@gmail.com (T.B.); 4 National Medical Research Center of Endocrinology, 117292 Moscow, Russia; nadshul@gmail.com; 5 Branch of the V. Serbsky National Medical Research Centre for Psychiatry and Narcology, 127994 Moscow, Russia; damulin_igor@mail.ru

**Keywords:** bile acids, metabolism, regulation of bile acids, bile salts

## Abstract

Bile acids are important physiological agents required for the absorption, distribution, metabolism, and excretion of nutrients. In addition, bile acids act as sensors of intestinal contents, which are determined by the change in the spectrum of bile acids during microbial transformation, as well as by gradual intestinal absorption. Entering the liver through the portal vein, bile acids regulate the activity of nuclear receptors, modify metabolic processes and the rate of formation of new bile acids from cholesterol, and also, in all likelihood, can significantly affect the detoxification of xenobiotics. Bile acids not absorbed by the liver can interact with a variety of cellular recipes in extrahepatic tissues. This provides review information on the synthesis of bile acids in various parts of the digestive tract, its regulation, and the physiological role of bile acids. Moreover, the present study describes the involvement of bile acids in micelle formation, the mechanism of intestinal absorption, and the influence of the intestinal microbiota on this process.

## 1. Introduction

Bile production is an important physiological function providing a pathway for the release of endo- and xenobiotics and lipid absorption from the intestinal lumen [1,2]. Bile secretion occurs as a result of the osmotic filtration of water through tight intercellular contacts [2]. Actively secreted components include bile acids, cholesterol and phospholipids, glutathione and various organic ions, bicarbonates, and immunoglobulins A and M [2,3]. Bile acids are synthesized in hepatocytes and secreted into the intestinal tract playing a crucial role in dietary-fat absorption. Another substantial function of bile acids is the control of intestinal microbial growth [4]. As the biological functions of bile acids in the small intestine include being ionized, the term «bile salts» is often used in publications. Recent studies demonstrated that bile acids are important metabolic regulators of glucose and lipid metabolism, energy homeostasis and xenobiotics excretion. The base of the bile acid molecule is C24-5β-cholanic acid, having a significant structural similarity to important steroid regulators, i.e., corticosteroids, mineralocorticoids, sex hormones, neurosteroids, and vitamin D. All of them are synthesized from a common precursor—cholesterol [5].

The biological functions of bile acids are realized through the activation of nuclear and cell-surface receptors, and probably through their influence on cellular membranes.

The disturbed homeostasis of bile acids, as a result of genetic mutations responsible for the synthesis and transport of these molecules, impaired hepatocytic function or intestinal transformations, may be associated with gallbladder disease, intrahepatic cholestasis, glucose intolerance and alcoholic and non-alcoholic fatty liver disease [4]. The present review discusses some aspects of the biological actions of bile acids that determine the prospects for their clinical applications, and which therefore need to be known. An assessment of the bile acid spectrum as a functional marker in intestinal and liver diseases and the potential use of bile acid receptor agonists/antagonists for targeting metabolic functions, in particular, lipid turnover and xenobiotics detoxication, is necessary; another important perspective is the peristaltic, secretion and permeability control. The possible negative effects associated with the pleiotropism of bile acid action should be taken into account. An interesting aspect, although not yet well studied, is the contribution of bile acids to the function of the central nervous system.

The primary analysis in this study is performed using a text-mining tool to highlight and select concepts from the PubMed ScanBious source (https://cryptome.ru/, accessed on 25 November 2021) [6,7]. Additionally, we analyze the literature from the past ten years and use secondary literature sources. The search is conducted using resources, such as the National Library of Medicine (PubMed) and Mendeley for the following keywords: “bile acids”, “metabolism bile acids”, “digestion”, “bile acids cell transport”, “regulation”, and “mutations of genes”.

## 2. Bile Acids in the Intestine

### 2.1. Bile Acid Synthesis in the Liver

**Figure 1:**
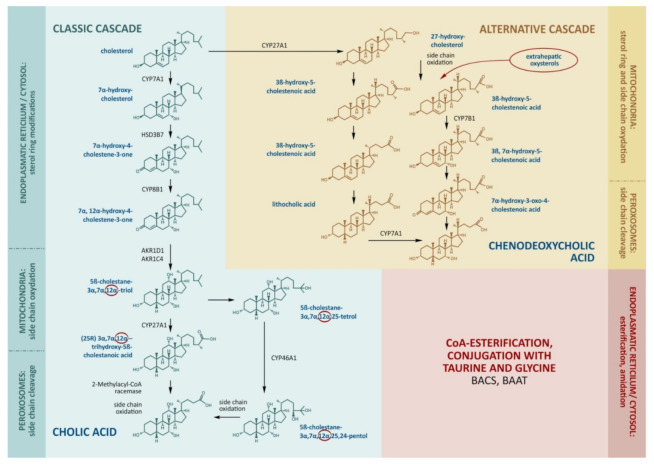
The main stages of cholesterol transformation and the production of bile acids. The key enzymes that determine the rate of bile acid formation include CYP7A1 and CYP8B1 in the classical cascade. CYP27A1 catalyzes side-chain oxidation in the classical cascade and initiates the alternative cascade. Cholic acid, chenodeoxycholic acid, and deoxycholic acid enter the bloodstream through the portal vein, inhibit CYP7A1, CYP8B1, and CYP27A1, and stimulate the enzymes for the conjugation of bile acids by BACS (bile acid–CoA synthase) and BAAT (bile acid–CoA:amino acid *N*-acyltransferase).

The production of bile acids consumes about half of the daily turnover of cholesterol. The main site of bile acid production is the hepatocyte, where its synthesis proceeds along the classical (neutral) and alternative (acidic) pathways (Figure 1).

The molecular cascades involve about 15 enzymes [8]. In the classical cascade, the initial stages take place in the cytoplasm and endoplasmic reticulum. In the first step, cholesterol is converted to 7α-hydroxycholesterol by cholesterol-7α-hydroxylase (CYP7A1). 

The alternative pathway is initiated by the mitochondrial enzyme sterol-27-hydroxylase (CYP27A1), which catalyzes side-chain hydroxylation. This is followed by the hydroxylation of ring B by oxysterol-7α-hydroxylase (CYP7B1), epimerization, a reduction in the double chain in ring B, and the shortening of the side chain by 3 carbon atoms. The end-product of the alternative pathway is chenodeoxycholic (3α,7α-dihydroxycholanic) acid (CDCA). CYP27A1 seems to be the key enzyme for the alternative cascade.

The alternative cascade utilizes the oxidized forms of cholesterol (oxysterols) formed in the liver and extrahepatic tissues as primary sources. CYP27A1 is expressed in the endothelial cells and macrophages, and the resulting 27-hydroxycholesterol can be transported to the liver in lipoproteins. The everyday hepatic 27-hydroxycholesterol absorption is nearly equal to the that produced outside the liver, hence the formation of CDCA may represent an important way of removing cholesterol from the circulation, endothelium, and macrophages [9,10,11]. Small cascades of cholesterol hydroxylation are described, involving endoplasmic reticulum enzymes, cholesterol 25-hydroxylase (in macrophages, the liver, and probably other tissues), and cholesterol-24-hydroxylase (in the brain). Under physiological conditions, these small cascades account for 5–16% of all cholesterol hydroxylation being rather notable substrate suppliers for the synthesis of bile acids [12,13]. In relation to tissue damage, peroxidation, inflammation, and catabolic processes, the contribution of oxysterols to the synthesis of bile acids may become more significant [14,15]. Moreover, oxysterols can be ingested in dietary animal fat and can be accumulated in improperly stored and cooked foods as a result of spontaneous cholesterol oxidation [15]. 

CA and CDCA are referred to as primary bile acids in humans, while, in mice, these are referred to as CA and β-muricholic acids [16,17]. Due to the hydroxylation of the steroid ring, they acquire amphipathic properties with hydrophobic (β) and hydrophilic (α) surfaces [5]. Synthesized CA and CDCA in the endoplasmic reticulum form CoA esters by the bile acid–CoA synthase enzyme [1,17]. Esterification removes the propionic acid residue in the side chain. At the stage preceding the secretion of the bile acids into the bile, the side chain is conjugated with glycine and taurine by the bile acid–CoA:amino acid *N*-acyltransferase enzyme [8]. In the human liver, primary bile acids are amidated with glycine and taurine in a ratio of ≈3:1. The amide bond increases the bile acid ionization constant (pKa); those conjugated with glycine are characterized by pKa ≈ 3 and those with taurine, pKa < 2 (due to the sulfo group), while the pKa of unconjugated bile acid is ≈5. Reduced pKa provides bile acid solubility in the presence of hydrochloric acid [9,18].

In adults, the classical pathway is normally the predominant route of bile acid synthesis, and 90% of the pool of newly synthesized bile acids is represented by CA, while no more than 10% by CDCA [9]. In children, the alternative path plays a more significant role [11]. During fetal development, CYP7A1 is not yet expressed. That is why, in neonates, CDCA is a dominating bile acid in bile, except in monohydroxylated bile acids [9,18,19]. In cases of decreased CYP7B1 activity, neonates develop severe cholestatic liver disease [19].

### 2.2. Micelle Formation

In the lumen of the small intestine, bile salts are present in micellar concentrations and form mixed micelles with dietary lipids and their digestion products, such as monoacylglycerols and fatty acids [20]. Bile acids also solubilize non-polar lipids, such as cholesterol and fat-soluble vitamins, increasing their water solubility and facilitating their diffusion through the unstirred water layer for delivery to the intestinal epithelium [21]. Side-chain amidation increases the ability of bile salts to dissociate in the acidic environment of the stomach, protecting against precipitation in the presence of calcium. The ability of bile salts to form micelles is referred to as a more pronounced “separation” of hydrophilic and hydrophobic parts (amphipathicity). Bile salts with hydroxy substituents on both sides of the steroid nucleus are non-amphipathic and do not form micelles, and the side-chain shortening causes an exponential increase in the critical micellar concentration [22,23]. The salts of primary bile acids have distinct amphipathicity and exhibit a detergent action to form micellar structures with cholesterol and phospholipids. Micelles ensure the fluidity of bile; «trapping» hydrophobic molecular parts reduces the damaging effect of bile on the apical membranes of hepatocytes and the epithelium of the bile ducts. CA and CDCA both have clearly separated hydrophilic and hydrophobic regions in the steroid skeleton. However, the anion of CDCA has more pronounced detergent properties than the anion of cholic acid, i.e., CDCA has a greater tendency towards micelle formation due to a larger hydrophobic surface. 

Conjugation with taurine and glycine increases the amphipathic properties of bile salts, their negative charge and the ability to form single-layer bubbles and micelles. The water-enriched mucin of the small intestine allows micelles to penetrate the brush border, where lipids are protonated due to weakly acidic pH (provided by the Na^+^/H^+^ exchanger) leading to micellar disintegration. Lipids are captured by enterocytes via membrane fusion, pinocytosis or specific receptors, while the salts of conjugated bile acids are released [24]. Bile salts cannot be absorbed in the proximal and middle parts of the small intestine as there are no specific bile acid transporters, while passive absorption is hindered by their pronounced amphipathic properties and the presence of an electric charge [20,25]. Thus, bile acid amidation helps to maintain their high concentration in intestinal lumen and to effectively perform fat emulsification. The ability of bile salts to form micelles seems to play an important role in non-biliary transintestinal cholesterol clearance, which is partly mediated by transporter proteins encoded by the ABCG5/8 genes (ATP-binding cassette subfamily G member 5/8 (ABCG5/8) and is crucial for fecal cholesterol excretion [26,27,28].

### 2.3. Absorption

As they travel to the distal ileum, approximately 15% of bile salts undergo deconjugation by intestinal bacteria, and the glycine and taurine released are absorbed by micro-organisms [20]. The absorption of bile lipids begins in the proximal and mid-gut, while bile salts are absorbed mainly in the distal small intestine (ileum), and only about 5% of intestinal bile acids escape reabsorption and are eliminated in the feces. The bile salt molecule is too large to pass paracellularly through the tight junctions of the intestinal epithelium and must be taken up transcellularly by passive or active mechanisms [26]. However, since most of the pool of bile salts is conjugated and ionized taurine or glycine, their uptake across the apical brush-border membrane requires the presence of a transporter, and the active transporter-mediated absorption of bile salts is limited to the ileum [20].

Up to 95% of deprotonated primary bile acids are absorbed, with an Na+-dependent apical bile salt transporter (ABST) expressed on the ileal epithelium [20]. The passive absorption of protonated, uncharged, unconjugated bile acids and a small fraction of glycine conjugates occurs along the entire length of the intestine. Protonation occurs due to a weak acidic micro-environment in the thin unstirred liquid layer covering the brush border [20,27]. Bile salts are transferred in portal circulation with an organic ion transporter (organic solute transporter α/β), as well as the multidrug resistance protein 3 expressed on the basolateral membrane of enterocytes [20]. Peptide YY production, ileocecal junction, and the intrinsic effect of bile salts on the mucous membrane of the terminal part of the small intestine seem to display the “ileal brake mechanism” and promote their ileal absorption [28].

### 2.4. Microbial Transformations

Bile salts not absorbed in the ileum (normally ≈ 5%) enter the colon, an environment rich in micro-organisms, where they are modified by microbial enzymes. In the first stage, the final cleavage of glycine and taurine residues occurs by microbial bile salt hydrolases expressed mainly by anaerobic genera *Bacteroides*, *Clostridium*, *Lactobacillus*, and *Bifidobacteria*. The next stage is ring B dehydroxylation by 7α-dehydroxylase, mainly expressed by *Clostridium* and *Eubacterium* [29]. As a result, secondary acids are produced: deoxycholic acid (DCA) and lithocholic acid (LCA) from CA and CDCA, respectively. Secondary bile acids are characterized by low amphipathicity [30]. Despite the higher ability of such molecules for passive diffusion through cell membranes, secondary bile acids are normally not significantly absorbed in the colon, as the colonic epithelium has inherited a resistance to bile acid penetration, in contrast to hepatocytes and enterocytes. About half of DCAs and only a small part of LCAs can be passively absorbed in the colon normally [20]. Of great interest is the problem of increased absorption in abnormal intestinal permeability.

Other bile salt modifications under the influence of the intestinal microbiota were also observed. *Bacteroides, Eubacterium*, and *Lactobacillus* can esterify the molecules, while *Clostridium*, *Fusobacterium*, *Peptococcus*, and *Pseudomonas* can desulfate them. *Bacteroides*, *Clostridium*, *Escherichia*, *Eggerthella*, *Eubacterium*, *Peptostreptococcus*, and *Ruminococcus* are involved in the oxidation and epimerization of hydroxyl groups in the 3rd, 7th and 12th positions of the sterol ring [31,32]. Thus, the salts of secondary bile acids can be modified into various other forms, in particular, dehydroxy-, chio-, diketo-derivatives [33]. In particular, ursodeoxycholic (UDCA) is formed from CDCA. UDCA and its glycine and taurine conjugates constitute up to 5% of the circulating bile acid pool.

Thus, under physiological conditions, DCA, conjugated/deconjugated CA, and CDCA, constitute a fairly large proportion of all bile acids entering the liver via portal blood, with the approximate ratio of CA:CDCA:DCA 1:1.34:1 [34]. Smaller proportions are represented by LCA, UDCA, and various derivatives of DCA. 

Unlike other steroid derivatives, the metabolic pathways of the reverse conversion to cholesterol have not been described for bile acids, hence, the excretion of bile salts via the feces serves as one of the pathways for excretion of cholesterol from the body (~200 mg per day). The spectrum of bile acids in bile and feces is significantly different, with a high predominance of primary conjugated bile acids in bile and deconjugated/sulfated secondary bile salts in feces. 

### 2.5. Bile Acids in the Gallbladder and Extrahepatic Bile Ducts

The highest physiological concentration of bile acids is observed in the gallbladder (≈300 mmol/L), followed by the bile ducts (20–50 mmol/L) and intestines (≈10 mmol/L) and in blood serum (0–6 µmol/L) [35]. Therefore, a high bile concentration in the gallbladder occurs due to its pronounced ability to reabsorb water and electrolytes. The composition of gallbladder bile is not well studied due to the difficulties in obtaining samples for analysis. CDCA more efficiently forms mixed micelles with cholesterol and phosphatidylcholine and, therefore, the CA/CDCA ratio may affect the risk of gallstone formation. 

Due to the secretion of bicarbonates on the surface of the biliary epithelium, a “bicarbonate umbrella” is created, which protects against the aggressive action of weakly acidic bile acids. The alkaline reaction, depending on the activity of the secretion of the cystic fibrosis transmembrane conductance regulator, seems to be important to maintain the fluidity of bile.

Similar to enterocytes of distal ileum, biliary epithelium expresses apical bile salts absorbing transporter ABST and the exporting transporter organic solute transporter α/β on the basolateral membrane [36].

## 3. Enterohepatic Circulation and Bile Acid Cell Transport

The enterohepatic circulation of bile acids is carried out by the passive and active absorption of primary bile acids in the distal parts of the small intestine (and partially by the passive absorption of DCA in the large bowel). The biological functions of enterohepatic circulation seem not to be related, only to reverse regulation of the de novo synthesis of primary bile acids and the elimination of cholesterol, but also to the humoral regulation of physiological functions in extrahepatic functions, such as satiety, and the metabolism of energy in the adipose and muscle tissue. Enterohepatic circulation may present the role of bile acids as peculiar sensors of the intestinal environment. 

While the synthesis of bile acids, de novo, is approximately 5 g per day, their loss is approximately 0.05 g in feces and approximately 0.05 g in urine [37]. Cycles of the enterohepatic circulation of bile acids occur 2–4 times a day [37]. The highest levels of bile acids in the systemic circulation are observed, on average, 1.5–2 h after a meal. The relative contribution of various factors to the plasma concentration of bile acids was studied. The propulsive activity of the intestine increases their plasma concentration 3 times 45 min after a meal. The contraction of the gallbladder further increases the concentration of plasma bile acids 90–120 min after a meal and with a recovery to baseline 360 min later [38]. A biological model of bile acid plasma concentration was constructed. During fasting, the plasma concentration of bile acids depends on the rate of primary bile acids synthesis, the passive and active absorption in the ileum, and the rate of distal intestinal transit. After a meal, the plasma concentration of bile acids is significantly affected by the proximal intestinal transit [38].

The absorbed bile acids enter the liver through the portal vein, where they are captured by hepatocytes from the side of the base-lateral membrane. Here, sthe odium taurocholate co-transporting polypeptide (NTCP) captures conjugated bile acids. NTCP absorbs approximately 80% of the bile acids entering the hepatocyte [39,40]. Unconjugated bile acids are captured by organic anion-transporting polypeptide 1B1/1B3 located on the base-lateral membrane [35]. Bile acids with minimally expressed polarity (in particular, LCA) can be captured by fusion with the hepatocyte membrane. This mechanism is probably important to limit the entry of potentially toxic bile acids into the systemic circulation. In hepatocytes, deconjugated bile acids again bind with glycine and taurine and are excreted into the bile. LCA acid undergoes sulfation, which dramatically reduces its ability for subsequent passive intestinal absorption and promotes its excretion (feces). Thus, the sulfation of LCA is the most important stage in the excretion of LCA having the highest toxic potential.

The secretion of bile acids into the bile is one of the most important factors that determines their movement along the bile ducts and further bile formation [2]. The main carrier of bile acids from the hepatocytes to bile is the bile salt exporting pump expressed on the apical membrane (BSEP) [41]. Bile acid secretion can also be mediated by multidrug resistance protein 2 (or ABCC2 or cMOAT). Multidrug resistance protein 2 also secretes conjugated bilirubin and, in mice, this pathway has been shown to unload the hepatocyte of excess bile acids in cholestasis to some extent, while it is of little value in humans [5,8,36]. Together with a portion of the apical membrane and adjacent tight contacts, the bile salt exporting pump and multidrug resistance protein 2 constitute the so-called secretory compartment of the hepatocytes, the function of which is greatly dependent on the cytoskeleton [2]. The secretion of bile acids and other components of bile by hepatocytes and cholangiocytes is an adenosine triphosphate-dependent process having high energy costs [2].

In cholestasis, other export pumps of the hepatocyte basolateral membrane also play an auxiliary role in bile acid elimination from the hepatocyte. These are, for example, proteins associated with the multidrug resistance-associated protein 3/4 with the highest affinity for more polar bile acids (CA, glyco-CA, CDCA) and an organic solute transporter α/β [36,42]. Under physiological conditions, multidrug resistance protein 3 and multidrug resistance protein 4 are characterized by a low expression. Multidrug resistance protein 4 carries out the glutathione-dependent transfer of various conjugated steroids and may be of more importance for bile acid export in cholestasis [43,44,45]. In this case, the activity of multidrug resistance protein 4 increases at the transcriptional and post-transcriptional levels under the influence of primary bile acids, relatively higher CA concentrations and lower CDCA concentrations [46]. 

Bile acid transporters have also been found in other organs and tissues, indicating their signaling role outside the liver and intestines. In particular, multidrug resistance protein 4 was found in proximal renal tubules and the ependyma of the choroid plexus [43]. The blood–brain barrier expresses the carriers of bile acids typical for hepatocytes, biliary and intestinal epithelium, such as the bile salt exporting pump, ABST, multidrug resistance protein 3/4, and the organic anion-transporting polypeptide [47,48].

## 4. The Regulation of Bile Acid Synthesis

Bile acids are also signaling molecules and inflammatory agents that rapidly activate nuclear receptors and cellular signaling pathways that regulate lipid, glucose, and energy metabolism. In the liver, bile acids activate a nuclear receptor, the farnesoid X receptor (FXR), which induces an atypical partner, a small heterodimer of the nuclear receptor, which subsequently inhibits nuclear receptors, liver-related homologue-1, and hepatocyte nuclear factor 4-alpha, leading to the inhibition of transcriptions of the critical regulatory gene for bile acid synthesis, CYP7A1 and CYP8B1 [49,50,51]. In the gut, FXR induces the gut hormone, fibroblast growth factor 15 (FGF15; or FGF19 in humans), which activates hepatic FGF receptor 4 (FGFR4)-signaling to inhibit bile acid synthesis.

Conjugated bile acids are secreted into bile and stored in the gallbladder. Some bile acids enter the sinusoidal bloodstream and are reabsorbed as they pass through the renal tubules in the kidneys and circulate back to the liver via the mesenteric and arterial circulation. Some bile acids secreted in the bile ducts are reabsorbed in cholangiocytes and returned back to hepatocytes (cholangiohepatic shunt). After each meal, the contraction of the gallbladder ejects bile acids into the intestinal tract.

Of great importance is the activation of bile acid synthesis through the mechanism of nuclear receptor liver X receptor (LXR)-stimulation by oxysterols.

An important protective mechanism is that the suppression of CYP8B1 shifts the cascade of reactions towards the formation of CDCA and reduces the proportion of CA. In addition, glucocorticosteroids can influence the recirculation of bile acids.

### 4.1. The Mechanism of the Regulation of the Classical Cascade Involving the Farnesoid X Receptor

The farnesoid X receptor (FXR), or bile acid receptor, is expressed in various tissues, but mostly in ileal enterocytes and hepatocytes. FXR agonists are bile acids, and of them, CDCA has the highest affinity. The two main ways in which FXR regulates bile acid production are described. 

The first pathway involves the interaction of bile acids with the FXR of ileal enterocytes, leading to the production of the endocrine peptide fibroblast growth factor 19 in humans or fibroblast growth factor 15 in mice [52]. Entering the portal blood and hepatocytes, fibroblast growth factor 19 interacts with the complex “fibroblast growth factor 4 receptor—β-klotho co-receptor”. This results in the suppression of the CYP7A1 gene expression and decreased CA production. FGF15 and FGF19 share only a 53% amino acid identity [53]. In humans, serum FGF19 levels peak 1.5–2 h after postprandial bile acid release [54], and induced FGF19 suppresses bile acid synthesis. FGF19 levels are reduced in subjects receiving cholestyramine, a bile acid sequestrant [54]. The overproduction of bile acids causes bile acid diarrhea and reduces the level of circulating FGF19 [55].

Intracellular signals causing the suppression of CYP7A1 are not well understood; it is likely that the Src homology 2 (SH2)-containing protein tyrosine phosphatase 2 plays a role in this [56]. Thus, this important mechanism of negative feedback between the content of bile salts in the ileum and the synthesis of bile acids, de novo, contributes to the maintenance of a constant pool of bile acids. The intravenous infusion of bile acids is not accompanied by such effects [57]. β-klotho increases the sensitivity of hepatocytes to the signaling effect of fibroblast growth factor 19. Defects in the expression of fibroblast growth factor 4 and β-klotho can disrupt the negative feedback mechanism. The rs17618244 (G > A) polymorphism of the β-klotho gene and rs1966265 (G > A) of the fibroblast growth factor 4 gene are associated with irritable bowel syndrome with accelerated transit, and the rs17618244 (G > A) polymorphism of the β-klotho gene is associated with the hepatocytes ballooning and necrosis reflecting increased lipotoxicity [58,59]. The regulatory pathway involving FXR and fibroblast growth factor 19 may be impaired in ileal disease. Fibroblast growth factor 19 can also be produced by biliary epithelial cells; in subhepatic cholestasis, this feedback mechanism appears to protect hepatocytes from bile acid damage [60]. 

The second pathway involving a feedback mechanism by FXR takes place in the liver tissue. In cholestasis, this mechanism acts as a protective against the damage of hepatocytes with a high bile acid concentration [61]. In the hepatocyte, CDCA interacts with FXR, and FXR moves to the nucleus, where it forms a heterodimeric complex with the retinoid X receptor, which binds to hormone-responsive DNA elements [62]. The result is the production of a small heterodimer partner, which, in turn, suppresses the transactivation of hepatocyte nuclear factor 4α and, ultimately, the expression of CYP7A1 and CYP8B1 and the production of CA and CDCA [11,40]. The inhibitory effect of the small heterodimer partner also occurs through the suppression of liver receptor homolog 1 [63]. In addition, the small heterodimer partner suppresses the transcription of the sodium taurocholate transporter gene, reducing the uptake of bile acids by hepatocytes from the bloodstream [64]. 

CYP8B1 gene expression is regulated by the fetoprotein transcription factor (FTF) and hepatocyte nuclear factor 4α (HNF 4α), with FTF likely playing a more important role. The small heterodimer partner-mediated repression of CYP8B1 transcription is mediated by both FTF and HNF 4α. When the small heterodimer partner binds FTF and/or HNF 4α, the resulting complex is inhibitory and represses CYP8B1 transcription [51,65].

In addition to the aforementioned effects, FXR indirectly induces the expression of the bile acid–CoA synthase and bile acid–CoA:amino acid *N*-acyltransferase genes and promotes the activity of the export pump of bile acids supporting the excretion of bile acids from hepatocytes into bile. The effect on the activity of other reserve bile acid exporters has also been described in cholestasis: multidrug resistance protein 2 on the apical membrane of hepatocytes and cholangiocytes (at the same time, the export of bilirubin is enhanced), multidrug resistance proteins 3 and 4, the organic solute transporter α/β, and the organic anion-transporting polypeptide 1B3 on the base-lateral membrane (at the same time, the excretion of phospholipids and xenobiotics is enhanced). In cholangiocytes and the ileum, the FXR, through the small heterodimer partner, suppresses the absorption of bile acids [64,66].

Bile acids bind FXRs with varying affinities. Lew et al. reported the binding affinity of several bile acids for human FXRs [67]. The binding affinity from high (left) to low (right) was found to be lithocholic acid (LCA) > CDCA > Tauro CDCA > Glyco CDCA ≫ DCA > ursodeoxycholic acid (UDCA) ≫ CA > Tauro CA > Glyco CA.

There seems to be no definite mechanism for the reverse regulation of the alternative cascade of bile acid synthesis. Its activity is influenced by one of the mechanisms regulating the transfer of cholesterol into mitochondria and functioning in the so-called steroidogenic tissues, including the liver and brain. One of the important mechanisms involves the steroidogenic acute regulatory protein, which can promote the activation of an alternative pathway with CDCA synthesis [68]. Steroidogenic acute regulatory protein activity is increased under the influence of cAMP, alcohol, and some exogenous substances [69]. Low steroidogenic acute regulatory protein activity contributes to the development of the fatty degeneration of the liver by reducing cholesterol utilization [70].

The oncogene of avian musculoaponeurotic fibrosarcoma and avian musculoaponeurotic fibrosarcoma oncogene homolog G (MAFG), the production of which increases under the influence of FXR and in adenovirus infection, can suppress the transcription of genes of the main enzymes of bile acid synthesis. The FXR/MAFG linkage suppresses the expression of several key enzymes of the classical and alternative cascades of bile acod synthesis, such as CYP7A1, CYP8B1, CYP27A1, and CYP7B1. The degree of their suppression may influence the ratio of bile acids in the general pool [61].

### 4.2. Biliary Tract in the Regulation of Bile Acid Synthesis

Previously, the cholehepatic shunt pathway has been proposed to explain the hypercholeretic nature of some bile acids [71]. This hypothesis suggested that bile acids in their protonated, uncharged form undergo passive biliary absorption, followed by the transfer of bile acids back to hepatocytes for re-secretion into bile. More recent studies have shown that, in the presence of bile duct obstruction, bile acids can enter cholangiocytes [71]. After the discovery of the expression of the bile acid transporter on the apical membrane of cholangiocytes, ASBT5-8, there has been renewed interest in the cholehepatic shunting of bile acid. Studies have shown that the adaptation of the mechanisms for the uptake of apical bile acid by cholangiocytes occurs both under physiological and pathophysiological conditions [72,73]. Other studies have shown that bile acid efflux mechanisms (ASBT and multidrug resistance transporter 3) are present on the basolateral membrane of cholangiocytes, providing a pathway for bile acids to enter the circulation [74,75].

The gallbladder is likely of great importance in the negative feedback mechanism of the regulation of bile acid synthesis. In the epithelium of the gallbladder, the content of fibroblast growth factor 19 mRNA is 250 times higher than that in the distal ileum, and the concentration of fibroblast growth factor 19 in gallbladder bile is 23 times higher than in serum. The expression of fibroblast growth factor 19 in the gallbladder is induced by an increased concentration of CDCA as the most potent FXR agonist [2].

### 4.3. Other Factors Affecting the Regulation of Bile Acids

Members of the LXR family are presented by LXRα and LXRβ. LXRα is highly expressed in the liver (both in humans and mice) and has a stronger affinity for the LXR response element (LXRE) binding to the CYP7A1 gene promoter [76]. Oxysterol-activated LXRα, in combination with liver receptor homolog 1, induces CYP27A1 transcription, bile acid synthesis, and thus indirectly increases cholesterol excretion. Factors affecting the activity of CYP7A1 also include genetic polymorphism in the CYP7A1 gene, hepatitis B virus infection (virus binding to the cellular receptor leads to decreased CYP7A1 expression), and vitamin C content (necessary for the regeneration of iron in the cytochrome) [77,78]. 

The isolated suppression of CYP8B1 shifts the cascade of the reactions towards the formation of CDCA and reduces the proportion of CA; however, the total size of the bile acid pool does not change [61]. The transcription of the CYP8B1 gene may be inhibited in inflammation under the influence of interleukin-1β acting through the mitogen-activated protein kinase/c-Jun N-terminal kinase pathway, which, in turn, suppresses the expression of the hepatocyte nuclear factor 4α gene and its ability to interact with DNA. This mechanism may play an important role in protecting hepatocytes in inflammation and cholestasis [79]. 

Glucocorticoids can significantly affect the recirculation of bile acids; they stimulate the ileal absorption of bile acids by increasing the expression of ABST and increase the uptake of bile acids by hepatocytes by increasing the expression of sodium taurocholate co-transporting polypeptides. The expression of CYP7A1 and the corresponding CA synthesis may be reduced in this case. Such changes are also accompanied by an increase in the bile acid content in the blood serum and a decrease in bile salt content in the feces [80].

## 5. Extensive Physiological Role of Bile Acids

### 5.1. Bile Acid Receptors

Some bile salts remain in the systemic circulation and can exert physiologic effects in other organs and tissues. Blood contains about 28 derivatives of bile acids, and the most polar (CA, CDCA), as well as DCA, are more prevalent [81].

Receptors that interact with bile acids are localized in various organs and tissues, and these various membranes and nuclear receptors are described in Table 1.

More amphipathic bile acids interact with a high affinity with membrane receptors, particularly, G-protein coupled receptors. The activation of membrane receptors leads to rapid metabolic effects.

Less amphipathic bile acids interact with a high affinity with nuclear receptors, similar to steroid hormones. Most of the nuclear receptors have two main structural domains—ligand- and DNA-binding domains. The nuclear receptor–bile acid complex is immersed in the cytoplasm and migrates to the nucleus, where it modulates gene expression. Thus, delayed metabolic effects are observed. Bile acids with a higher hepatotoxic potential (in particular, LCA) also significantly interact with nuclear receptors that regulate the detoxification of xenobiotics (Table 1).

### 5.2. Bile Acids in Cholesterol Homeostasis

Bile acids play an important role in the metabolism of lipids. They are essential for the formation of mixed micelles in the small intestine, facilitating the solubilization, digestion, and absorption of dietary lipids and fat-soluble vitamins. 

The metabolism of bile acids is closely related to cholesterol homeostasis in the body; the conversion of cholesterol into bile acids and bile acid-stimulated secretion of cholesterol into the bile are both important ways of removing cholesterol from the body. 

In addition, the liver metabolizes cholesterol through dietary absorption, receptor-mediated uptake, and de novo synthesis. Intracellular cholesterol/oxysterols play an important role in the regulation of cholesterol synthesis via the transcription factor, sterol response element-binding protein 2 (SREBP2). While the level of intracellular cholesterol increases, the SREBP2 precursor (125 kDa) forms a complex with the insulin-inducible gene (INSIG) activating the cleavage of SREBP (SCAP), which is stored in the endoplasmic reticulum membrane. When the cholesterol level decreases, SCAP escorts the SREBP2 precursor to the Golgi apparatus, where two steroid-sensitive proteases (S1P and S2P) cleave the N-terminal fragment (68 kDa), subsequently moving into the nucleus to activate their target genes, including the genes of the low-density lipoprotein receptor and key genes involved in de novo cholesterol synthesis [105].

The role of LXR has been demonstrated in LXR-null (Lxrα^−/−^) mice. Since such mice did not exhibit a compensatory increase in bile acid synthesis due to the lack of CYP7A1 gene activation, a diet high in cholesterol was accompanied by the excessive accumulation of cholesterol in the liver and impaired liver function [76]. The accumulation of more cholesterol in the liver of LXR-null mice may also be due to the dysfunction of ABCA1, ABCG1, and ABCG5/G8, which are involved in cholesterol elimination and whose activity is also regulated by LXR.

The effect of LXRα on the transcription of the CYP7A1 gene in humans is less pronounced than in mice. This was determined by the transcriptional regulation of luciferase reporter gene expression under the control of the human CYP7A1 promoter in HepG2 cells, a model established to study the regulation of cholesterol 7α-hydroxylase [106].

### 5.3. Bile Acids in the Brain

The potential role of bile acids in brain physiology is an interesting field of research. The brain contains approximately one quarter of the total cholesterol in the body. The blood–brain barrier is impermeable for cholesterol in contrast to oxysterols having a hydroxylated side chain. This is the way to regulate the excess cholesterol removal from the central nervous system. The daily amount of 24-hydroxycholesterol leaving the brain and entering the venous blood is very similar to its absorption by the liver. A half of this amount, after conjugation with sulfuric and glucuronic acids, is excreted to bile [107]. Another half of 24-hydroxycholesterol undergoes 7α-hydroxylation and serves as a source of bile acid formation [108,109]. Thus, the metabolism of brain cholesterol may be closely related to the exchange of bile acids and bile composition. Under physiological conditions, the contribution of 24-hydroxycholesterol to the production of bile acids is not noticeable [110]. On the contrary, it seems relevant to study this “brain-hepatic aspect” in neuropsychiatric diseases. 

Interestingly, approximately 20 types of bile acids have been found in the brains of rats under physiological conditions, which are predominantly primary bile acids CA and CDCA. Their brain concentration exceeds that in the peripheral blood by 10 fold and is several times higher than the local content of the neurosterode pregnenolone [111]. The results of studies evaluating the bile acid ratio are somewhat contradictory [111,112]. Similar results were obtained for the human brain [113,114]. The authors concluded that such a high content of primary bile acids in the central nervous system may indicate their local formation. CYP8B1 and CYP27A1 responsible for CDCA synthesis are expressed in the cells of the central nervous system. The highest CYP27A1 activity is observed in the cerebral cortex, hippocampus, cerebellum, and caudate nucleus [115]. The importance of central nervous system diseases associated with changes in the metabolism of bile acids and the proportional relationship between CA and CDCA must be studied further [116].

## 6. The Action of Bile Acids on Cell Membranes

Studies conducted in the 1980s and 1990s showed that high concentrations of bile salts are toxic to epithelial cells in the gastrointestinal tract, including hepatocytes and cholangiocytes [117]. The hydrophobic part of the bile acid molecules allows it to interact with cell membranes and disrupt their structural integrity and plasticity (fluidity). In an amphiphile, as the hydrophobic molecular surface increases at the expense of the hydrophilic molecular surface, its critical micellar concentration decreases and its membrane toxicity also increases. In accordance with that, the degree of the hydrophobicity of bile acids is as follows: glycine conjugates of CA, CDCA, DCA > taurine conjugates of CA, CDCA, DCA > LCA > DCA > CDCA > CA; the polarity of conjugated bile acids is higher (in particular, in taurine conjugates) [118]. For example, DCA can induce the lysis of hepatocytes at a concentration 10 times lower than that of CA [119].

Conjugated primary bile acids do not exhibit cytotoxic effects at concentrations close to physiological conditions. In the presence of phosphatidylcholine, cholesterol, the concentration of primary bile acids, which are freely present in the aqueous phase, sharply decrease, as they form single-layer bubbles and mixed micelles, in which the hydrophobic regions of the molecules are enclosed. This explains why the primary bile acids in the biliary tract and small intestine do not display cytotoxic effects. The ability of bile acids to form micelles also promotes the passive movement of phospholipids from cholangiocyte membranes [120]. Studies demonstrate that, when in contact with the bile epithelium, conjugated bile acids can also extract part of the plasma membrane enzymes (5′-nucleotidase, alkaline phosphatase, phosphodiesterase I). This may explain the content of some proteins in bile [119].

At a concentration close to the amount required for the formation of micelles, membrane damage is possible. A deficiency of phosphatidylcholine in bile can increase the content of primary bile acids, which are freely present in the aqueous phase (monomers) leading to the damage of cholangiocytes [121]. The secretion of HCO^3−^ by the biliary epithelium creates a protective bicarbonate umbrella on its surface, which neutralizes bile acids and reduces their ability to penetrate the cell surface [96]. Patients with primary sclerosing cholangitis are increasingly being detected as deficient in the activity of the cystic fibrosis transmembrane conductance regulator, which regulates the transport of bicarbonates, indicating the probable damaging role of bile acids in the pathogenesis of this disease [122].

At higher concentrations, bile acids can incorporate their hydrophobic part into cell membranes and disrupt the orientation of membrane lipids [123]. Experiments with artificial membranes demonstrated that, at low concentrations, bile acids mainly interact with the surface of the lipid bilayer, where their hydrophobic part can penetrate the membrane [124].

The membranes of different cells are variably permeable to bile acids. Experiments in cell cultures showed only a minimal uptake of ^14^C- and ^3^H-labeled bile acids by colonic cells and a significantly higher uptake by hepatocytes. In the present study, the degree of hydrophobicity does not directly correlate with cellular uptake [125], whereas other studies have shown that hydrophobicity definitely affects the ability of bile acids to move across cell membranes. Bile acids interact with certain membrane areas being in the gel-like and liquid phases, which is determined by the relative content of cholesterol and phospholipids in the membrane. Areas that are rich in cholesterol are characterized by an increased resistance to solubilization. It is likely that cholesterol interacts with bile acids that are more hydrophobic, sequestering them in certain areas of the membrane [123]. While cholesterol provides the membrane’s rigidity, bile acids, on the contrary, increase its fluidity. At high concentrations, bile acids form “clusters” in the membrane, causing the lipid bilayer to separate, while the more hydrophilic ends form pores through which water-soluble substances can penetrate. LCAs are likely to form hydrogen bonds with the main phospholipid component 1-palmitoyl-2-oleoyl-sn-glycero-3-phosphocholine. At a significant concentration, bile acids can provoke membrane solubilization.

Less polar bile acids (DCAs and LCAs) have a more pronounced exfoliating effect, while more polar CA and its conjugates exhibit minimal effects. The thick mucin layer present in the colon, normally, may limit the contact of DCAs and LCAs with colonocytes. The passive absorption of bile acids in the distal parts of the small intestine and biliary tract occurs by the mechanism of transmembrane translocation, but bile acids are not fixed in the membrane bilayer and do not cause its disorganization. The transmembrane translocation of CA occurs faster than that of CDCAs and DCAs; the translocation of unconjugated bile acid also comes more readily than that of conjugated bile acids [124,126].

The protective effect of the most hydrophilic UDCA on the cells of the gastrointestinal tract is shown in a series of works [127,128,129]. Experiments with synthetic membranes revealed that UDCA prevented the damaging effect of DCA on membranes, only in the presence of membrane cholesterol [123]. In vitro, UDCA prevents the DCA-induced apoptosis of HCT116 cells due to the stimulation of the Akt/PKB cascade [128].

## 7. Mutations of Genes Controlling the Cellular Metabolism and Transport of Bile Acids

### 7.1. Mutation in Genes Encoding Enzymes of the Classical and Alternative Cascades of Bile Acid Synthesis

A genetically determined decrease in CYP7A1 activity increases the risk of cholesterol gallstones. Genetic variations in CYP7A1 affect its expression, and thus may influence the risk of gallbladder cancer [130,131]. The carriers of mild and moderate mutations of the CYP7A1 gene have an increased risk of non-alcoholic fatty liver disease and coronary heart disease [132,133]. The level of glycemia can influence the transcription of the CYP7A1 gene through the epigenetic regulation of histone acetylation [134]. Animals experiments revealed that the complete absence of CYP7A1 activity can also lead to death in newborns [132].

Mutations in the CYP8B1 gene in humans have not been studied, however, the loss of function mutation in CYP8B1 in some animals is of special interest, as they exhibit the decreased intestinal absorption of cholesterol, normal secretion of glucagon-like peptide-1, and no impairment of glucose metabolism [135]. 

Various mutations of the CYP27A1 gene have been described, including those that cause decreased CYP27A1 activity and the accumulation of cholesterol and cholestanol in tissues, especially a significant accumulation in the brain (cerebral tendinous xanthomatosis). The development of diarrhea is often observed, probably pathogenetically associated with CDCA deficiency. The most common mutation is the substitution of arginine for cysteine at position 362 (Arg362Cys) [136].

### 7.2. Mutation of Genes Encoding Enzymes for the Esterification and Amidation of Bile Acids

Several mutations in gene-encoding enzymes have been described, but their prevalence and clinical significance are not well understood. Mutations in bile acid–CoA synthase are associated with the development of neonatal cholestasis and the malabsorption of fats and fat-soluble vitamins [137].

In the carriers of bile acid–CoA:amino acid *N*-acyltransferase mutations, unconjugated bile acids cannot be excreted into the bile by the bile salt exporting pump, and are transferred from hepatocytes to plasma by passive diffusion. The content of bile acids in serum and urine can be high, however conjugates with glycine and taurine are practically absent. The content of bile salts is considerably reduced in the intestine; its sulfates and glucuronides dominate in the feces [137].

The deficiency of bile acid–CoA:amino acid *N*-acyltransferase in combination with a defect in the gene encoding the protein 2 tight junctions is accompanied by the development of cholemia, pruritus, and fat malabsorption [138].

### 7.3. Bile Acid Transporter Defects

Missense mutations, synonymous mutations, and deletions of the bile salt exporting pump gene are associated with the development of cholestatic liver diseases, such as benign recurrent intrahepatic cholestasis type 2, progressive familial intrahepatic cholestasis type 2, intrahepatic cholestasis of pregnant women, cholelithiasis, and intrahepatic calculi [139,140,141,142,143,144]. 

Mutations in solute carrier family 10 member 1, encoding the structure of the sodium taurocholate co-transporting polypeptide, lead to the development of hypercholanemia with an increase in the content of conjugated bile acids. The definite phenotype of this deviation has not been described; itching and signs of cholestasis, and other symptoms of liver damage are not typical [145]. This defect may be partially compensated by the function of organic anion-transporting polypeptide 1B1/1B3, but may have other effects in concomitant diseases, such as the cholestasis of pregnancy [145,146].

ASBT is responsible for the active uptake of bile acids by the terminal ileum [147]. The major role of ASBT in the intestinal absorption of bile acids is supported by genetic evidence that determines that the targeted inactivation of ASBT abolishes the enterohepatic bile acid cycle in mice [148,149,150], and ASBT mutations in humans are associated with the development of malabsorption syndrome [151]. According to pharmacological studies, the administration of small molecular weight ASBT inhibitors to animal models reduced the absorption of bile acids in the intestine [152,153,154].

## 8. Effects of Bile Acid Sequestrants

Most studies investigated the effects of the sequestrants of bile salts (bile acid-binding resins), whose cholesterol-lowering actions are also associated with the restriction of the intestinal absorption of bile acids [155]. The sequestrants of the bile salts were originally used to treat hypercholesterolemia and the malabsorption of bile acids [155,156]. The violation of the enterohepatic circulation of bile acids, by blocking their absorption in the intestine, stimulates the hepatic synthesis of bile acids de novo from cholesterol. The cholesterol requirement of the liver is met by increasing the synthesis of hepatic cholesterol and the clearance of the plasma low-density lipoprotein [157]. Although the sequestrants of bile salts acting by this mechanism have not been widely used for the treatment of hypercholesterolemia, they have presented benefits in lowering plasma cholesterol levels and reducing the incidence of cardiovascular disease in some studies [158,159]. The sequestrants of bile salts have been shown to improve glycemic control [155,160,161], and treatment with them increases the incorporation of sterols and fatty acids into bile and feces and also leads to a change in lipid metabolism through excretion [162]. In addition, by blocking the apical uptake of bile acids in iliac enterocytes, the sequestrants block the activation of the FXR–fibroblast growth factor 15/19 pathway, which increases the expression of CYP7A1 in the liver, changing the composition of the pool of bile acids. In model studies using mice treated with the sequestrant of bile salts, it was hypothesized that the increased synthesis of bile salts via the CYP7A1 pathway increases the intake of natural Takeda G-protein receptor 5 agonists (CA and its derivatives) into the systemic circulation. This leads to an increase in energy expenditure in muscle and brown adipose tissues [162]. Blocking intestinal absorption also increases the transport of bile acids to the colon and may enhance the Takeda G-protein receptor 5 mediated release of glucagon-like peptide-1, resulting in increased insulin sensitivity [163,164]. In addition, the administration of ASBT inhibitors with a low molecular weight has effects similar to those described for the sequestrants of bile acids. For example, ASBT inhibitors reduced low-density lipoprotein cholesterol levels in various animal models [165,166].

The loss of ASBT or organic solute transporter α/β is known to impair the intestinal absorption of bile salts [159], however studies [167,168] in mice lacking ASBT and organic solute transporter α/β, important phenotypic differences in homeostasis of bile acids, have been identified, which may affect lipid and glucose metabolism. Mice lacking ASBT have a similar metabolic phenotype to that described for treatment with sequestrants of bile salts or ASBT inhibitors [169]. The inactivation of ASBT increases hepatic CYP7A1 and also reduces SREBP1c, improving the metabolism of triglycerides.

The abundance of ursodeoxycholic acid, rifaximin, cholestyramine has no effect on the level of bile acids in the blood serum. The effect of this defect on various diseases has not yet been studied. Plasmapheresis and the use of the ABST antagonist elobixibate are possible measures to reduce the level of bile acids in the blood, however, they need to be studied further [146].

## 9. Conclusions

The interest in bile acids in the human body is constantly increasing. This is due to the fact that they are involved in many physiological processes, the violation of which contributes to the formation of a wide range of hepatobiliary and intestinal pathologies. Despite the fact that bile acids share a similar chemical structure, they not only have a variety of physical properties, but also differ significantly in their biological characteristics. The main purpose of bile acids is well known—the participation in the digestion and absorption of fats. Studies have shown that the role of bile acids is not limited to their participation only in the processes of digestion. Their role in various pathological processes is obvious, both as an etiological factor and as mediators of individual links in pathogenesis. Genetically determined disturbances in their synthesis, biotransformation, and/or transport may result in the development of severe pathology.

However, based on the analysis of literary sources, it can be concluded that the role of bile acids in the development of pathological processes is still not completely clear and is the subject of further research. As knowledge about the physiological role of bile acids in the human body expands, new concepts will appear that explain the reasons for the emergence and formation of a number of pathological processes that, to date, are still unclear.

## Figures and Tables

**Table 1:** Major bile acid receptors.

Receptor	Functions	Localization	Ref.
Nuclear receptors			
Farnesoid X receptor	The main regulator of the enterohepatic circulation of BAs. Induces the production of FGF19, CYP3A4, and PXR in the ileum. Suppresses the transcription of the CYP7A1 and CYP8B1 genes and the synthesis of BAs. Suppresses the transcription of the NTCP gene and the uptake of BA by hepatocytes. Increases the activity of BACS, BAAT, BSEP, and MRP2 and simulates the export of BAs and bilirubin to bile. Suppresses ABST and OATP and the absorption of BAs by cholangiocytes by the ileal epithelium. Vasodilating action in the systemic and splanchnic circulation.	Epithelium of the ileum, hepatocytes, cholangiocytes, endothelium of sinusoids, renal epithelium, adrenal cortex, and cells of innate and adaptive immunity.	[82,83,84,85]
Nuclear receptor subfamily 1 group H member 3	Regulates the remodeling of the phospholipids of the endoplasmic reticulum, and affects the processing of SREBF1 and the inclusion of triglycerides in VLDL. Suppresses the stress of the endoplasmic reticulum and acute phase reactions. Reduces the absorption of cholesterol in the intestines. Increases the activity of CYP7A1 and the synthesis of Bas; promotes the transport of cholesterol from peripheral tissues to the liver and its transformation into BA. Activates sterol response element-binding protein-1c, regulating lipogenesis.	Hepatocytes, enterocytes, renal epithelium, adipose tissue, skeletal muscles, and cells of innate and adaptive immunity.	[86,87,88]
Vitamin D receptor	Modulation of the intestinal microbiota composition and indirect influence on the conversion of secondary BAs. Potential impact on the risk of developing colorectal cancer.	The ileum, endocrine glands, skin, cells of innate and adaptive immunity.	[33,89]
Nuclear receptors—xenobiotic sensors			
Constitutive activated receptor, nuclear receptor subfamily 1, group I, member 3	Many effects are mediated by the HNF4α transcription factor. Suppression of CYP7A expression and BA synthesis with an increase in the content of LCA in the blood; activation of phase II enzymes for the detoxification of xenobiotics (sulfotransferases, glucoronosultransferases, glutathione S-transferases), including the activation of LCA sulfation and bilirubin conjugation. Activation of transporters (MRP, MDR, and OATP). Suppression of gluconeogenesis, development of steatosis, and decrease in thyroxine activity.	Hepatocytes and renal tubular epithelium.	[90,91,92]
Pregnane X receptor, nuclear receptor subfamily 1, group I, member 3	The effects are similar to those of constitutive androstane receptor activation (mediated by the transcription factor HNF4α); CYP3A43 activation; suppression of the inflammatory cascade caused by the influence of NFκB and the maintenance of the intestinal epithelial barrier; suppression of CYP7A1.	Hepatocytes and intestinal epithelium.	[85,93,94]
Membrane receptors			
G protein–coupled bile acid receptor 1, Takeda G-protein receptor 5	Systemic effects of Bas; regulation of intestinal motility and metabolism; relaxation of the gallbladder during the interdigestive period (together with FGF19); vasodilating action in the systemic and splanchnic circulation; regulation of the proliferation of non-ciliated cholangiocytes, a possible role in the development of cholangiocellular cancer.	Epithelium of the ileum, cholangiocytes, smooth muscle cells, endothelium (in particular, the endothelium of sinusoids), adipose tissue, and cells of innate and adaptive immunity.	[83,95,96]
Sphingosine-1-phosphate receptor 2	Increased activity of enzymes of lipid metabolism (SREBP1c, FAS, LDLR, FXRα, and PPARγ) and glucose (ERK1/2 and AKT signaling pathways and glycogen synthesis); regulates the differentiation of endothelial cells; promotes the growth and metastasis of cholangiocarcinoma.	Hepatocytes, intestinal epithelium, endothelium, vascular smooth muscle cells, myocardium, and fibroblasts.	[97,98]
Muscarinic receptors M2, M3	Stimulation of intestinal motility, negative chronotropic action. Probably promote the growth of colon cancer.	Intestinal smooth muscle cells, exocrine glands, and myocardiu.	[99,100,101,102]
Vascular endothelial growth factor	Prevention of bile duct injury, possibly fibrosis. New vessel formation.	Cell lines of stomach and colon cancer.	[103,104]

AKT—protein kinase B; ABST—apical bile salt transporter; BAAT—bile acid–CoA:amino acid N-acyltransferase; BA—bile acid; BACS—bile acid–CoA synthase; BSEP—bile salt exporting pump; ERK1/2—extracellular signal-regulated kinase 1/2; FAS—fatty acid synthase; FGF—fibroblast growth factor; FXRα—farnesoid X receptor α; HNF4α—hepatocyte nuclear factor 4α; LCA—lithocholic acid; LDLR—low-density lipoprotein receptor; MDR—multidrug resistance transporter; MRP–multidrug resistance-associated protein; NF-kB—nuclear factor kappa-light-chain-enhancer of activated B cells; NTCP—sodium taurocholate co-transporting polypeptide; OATP—organic anion-transporting polypeptide; PPARγ—peroxisome proliferator-activated receptor-γ; PXR—pregnane X receptor; and VLDL—very low-density lipoprotein.

## Data Availability

This is a review paper that collected from public data listed in the “Reference” and from open access web-source PubMed.
